# Determinants Impacting User Behavior towards Emergency Use Intentions of m-Health Services in Taiwan

**DOI:** 10.3390/healthcare9050535

**Published:** 2021-05-03

**Authors:** Wan-I Lee, Hsin-Pin Fu, Nelio Mendoza, Tzu-Yu Liu

**Affiliations:** Department of Marketing and Distribution, National Kaohsiung University of Science and Technology, Kaohsiung City 824, Taiwan; wilee@nkust.edu.tw (W.-IL.); i107123110@nkust.edu.tw (N.M.); f108113109@nkust.edu.tw (T.-Y.L.)

**Keywords:** emergency use intention, m-Health, satisfaction, usage behavior

## Abstract

Emergency usage intention and behavior are crucial to business service success for m-Health providers and patient healthcare service. This study aimed to identify the factors that influence m-Health acceptance and the effect of emergency use intentions on usage behavior among Taiwanese m-Health users by adopting and extending the Unified Theory of Acceptance and Use of Technology (UTAUT). This study also examines the moderating role of gender and age in the effects of the independent variables on satisfaction with m-Health services. An online questionnaire was used to collect data from 371 participants. The results revealed that performance expectancy, facilitating conditions, and trust had positive effects on user satisfaction. Additionally, m-Health knowledge and user satisfaction had positive effects on emergency use intentions. However, social influence and effort expectancy did not have a significant effect on satisfaction. Moreover, age and gender significantly moderated the effects of some predictors.

## 1. Introduction

On 31 December 2019, the World Health Organization (WHO) was notified of pneumonia cases with an unknown etiology that had been detected in Wuhan in Hubei Province in China. When the outbreak was first reported in December, some experts predicted that Taiwan would witness the highest number of COVID-19 cases because of the high number of daily flights and geographical proximity between China and Taiwan [1]. However, on 28 February 2021, there were only 955 confirmed cases in Taiwan. Of these, 839 were imported, and nine deaths were caused by COVID-19 [2]. When compared to other countries that neighbor China, only a few infected cases were reported in Taiwan. The Taiwanese government developed a public health response mechanism based on prior experience gained during the 2003 severe acute respiratory syndrome (SARS) outbreak. Information technology tools, including m-Health, eHealth, QR code scanning, and online reporting of travel history, were used to help health officials identify and trace suspected patients and high-risk individuals [1].

In such contexts, technology plays an essential role in strengthening the health system of a society. Health information technology (HIT) improves individuals’ health statuses by improving the quality of health services and health management [3,4]. In recent years, mobile health (m-Health) technology has offered an alternative, which involves information and telecommunication technology (e.g., smartphones). The WHO has defined m-Health as the practice of public and medical healthcare through the use of mobile (e.g., smartphones, personal digital assistants) and other wireless devices. m-Health can efficiently support the healthcare system by reducing unnecessary healthcare waste in medical centers and delivering efficient healthcare. Waste refer as refers to a measure of inefficiency in different aspects such as billing and insurance functions, patients’ waiting time, inappropriate use of medical resources, inappropriate operation flow, and low-value outputs [5]. In this context, mobile health can increase the efficacy and convenience of care for patients and healthcare providers, reducing waste and supply costs during the COVID-19 pandemic.

According to the Taiwan National Communications Commission, the number of mobile phone connections (e.g., active SIM cards) was 29.31 million in 2019 [5]. More specifically in Taiwan, the total mobile phone subscriptions were recorded as 120% of total Taiwanese residents [5]. In recent years, m-Health has drawn the attention of Taiwanese hospitals and users [6]. In 2008, the Department of Health in Taiwan reported that the use of m-Health among medical professionals was only 16.3%. Further, only 13.7% of hospitals in Taiwan were using mobile phones to provide healthcare services [6]. However, mobile phones can be widely applied to help medical professionals complete their work safely and efficiently in the context of the COVID-19 pandemic (e.g., electronic patient records, real-time monitoring and mask distribution). This simply implies that there is a clear need and importance to explore m-Health emergency use intention during the Covid-19 pandemic.

The outbreak of COVID-19 has led to a public health emergency in several countries. It has necessitated changes in how individuals access healthcare resources during emergencies. To address this need, many countries have been providing m-Health and telehealth services (e.g., observing quarantine patients, providing healthcare information, distribution of medical masks, etc.) [1,7,8]. Moreover, health professionals have been promoting social distancing, which includes avoiding crowded hospitals and waiting rooms. Additionally, the Taiwanese government controlled the sale and distribution of masks during the early stages of the pandemic, masks were sold at a fair price, and distribution was even, to reduce the risk of COVID-19 transmission. In this manner, m-Health applications can help reduce the risk of COVID-19 transmission by promoting social distancing and facilitating physician-patient communication [1,8].

Kijsanayotin et al. [9] studied the motivational factor that influenced m-Health adoption in Thailand. By integrating the Unified Theory of acceptance and Use of Technology (UTAUT), they found that social influence, performance expectancy and effort expectancy positively influenced m-Health acceptance in Thailand. Hsiao et al. [10] investigated a model of m-Health technology acceptance by the elderly in Taiwan. Based on the technology acceptance model (TAM), they reported that perceived usefulness (i.e., effort expectancy) and health knowledge positively influenced the elderly’s intention to use m-Health services. To et al. [11] explored how perceived easy use (i.e., performance expectancy) and perceived usefulness (i.e., effort expectancy) affect users’ intention to use m-Health. Using responses from 486 chine young adults in China, they reported that effort expectancy and performance expectancy significantly influenced Chinese young adults’ intention to use m-Health. Most past studies in this domain have focused on routine use intentions [12,13,14,15], but very few have examined emergency use intentions [13]. Moreover, the factors (e.g., m-Health knowledge and satisfaction) that influence emergency use intentions toward usage behavior have rarely been investigated. Therefore, this study’s theoretical framework was designed based on the unified theory of acceptance and use of technology (UTAUT) [16]. This framework was adopted to examine the impact of m-Health use on Taiwanese users. The findings could offer helpful insights into infection control.

The primary purpose of this study was to investigate the effect of crucial factors extracted from the extended UTAUT model (performance expectancy, effort expectancy, social influence, facilitating conditions, and trust), the moderating effect of gender and age, and the effect of user satisfaction on m-Health usage behaviors through emergency use intentions during the COVID-19 pandemic in Taiwan. Additionally, this study aimed to examine the effect of m-Health knowledge on emergency use intentions and their effect on m-Health usage behaviors among Taiwan users. In this research model, m-Health was used to analyze mask distribution, medical appointments, and the mobile health consultation of patients in quarantine during the COVID-19 pandemic in Taiwan.

## 2. Literature Review and Hypothesis Development

### 2.1. m-Health in Taiwan

Over the past 20 years, the Taiwanese government has been implementing the National Information and Communication Development Plan, including the e-Taiwan, M-Taiwan, and u-Taiwan Plans. The objective is to promote, upgrade, and build the foundation of information and communications technology (ICT) by using new and innovative applications to improve the quality of life in Taiwan [17]. The government incorporated ICTs (e.g., mobile application services) into government and health delivery services (e.g., m-Health applications) for the benefit of the public [18]. During the SARS outbreak, the Taiwanese government formulated m-Health services exclusively reserved for hospitals, emergency ambulances, and older adults living alone [19]. In 2020, the Taiwanese government decided to standardize and redefine the COVID-19 pandemic Mask Map and devise novel strategies to help individuals purchase masks through mobile applications [20]. Additionally, the services of medical appointments, mobile health consultation of patients during quarantine increase the efficacy and convenience of care for patients and healthcare providers, reducing unnecessary healthcare waste and improving productivity in healthcare systems during the COVID-19 pandemic. Taiwan has made great improvement in the development and delivery of healthcare services during the Covid-19 pandemic. The Taiwanese government developed a public health response mechanism based on health information technology (HIT) that allowed to eliminate unnecessary healthcare waste by enhancing the efficient administration, provision, and production of healthcare services.

### 2.2. Hypotheses Development

The UTAUT is the most widely used model in studies on technology adoption. It synthesizes the determinants of use intentions and behaviors more effectively than other previously proposed technology acceptance models (TAM) [10,16,21]. Further, m-Health usage behaviors have been explored from a wide range of theoretical perspectives. Examples include the UTAUT model and its extended version UTAUT2, reasoned action (TRA), and TAM theory. The UTAUT model posits that behavioral intentions are influenced by four important determinants: performance expectancy, effort expectancy, social influence, and facilitating conditions [16,22]. The UTAUT also includes various control variables, which moderate this relationship (e.g., experience, age, gender, and voluntary) [16,22].

Following past findings on m-Health use embedded within the UTAUT literature [23,24,25,26], we speculated that user experience with m-Health adoption will be reflected in their overall satisfaction and emergency use intentions and that both these variables will be influenced by performance expectancy, effort expectancy, social influence, facilitating conditions, and trust. We also expected m-Health knowledge to directly affect emergency use intentions [27]. In addition, age and gender were expected to independently play a moderating role in m-Health use in emergencies [16]

Performance expectancy has been defined as “the degree to which an individual believes that using the Information System will help him or her to attain gains in job performance” [16] (p. 447). Within the context of m-Health, performance expectancy refers to the perceived effectiveness of m-Health services among users. Therefore, if users perceive m-Health services to be effective in improving their healthcare experience, they are likely to feel satisfied with the service, which will influence their use intentions. According to Bhattacherjee [24], if users perceive an information system as useful, they are likely to feel highly satisfied and motivated to continue using the system. Deng et al. [28] found that performance expectancy decreases user satisfaction when it carries hedonic value but increases user satisfaction when it carries utilitarian value. In other words, when m-Health services fail to satisfy user needs, they adversely affect user satisfaction with these services. Thus, within the context of the ongoing COVID-19 pandemic in Taiwan (i.e., an emergency), the following hypothesis was proposed:

**Hypothesis** **1** **(H1).** *Performance expectancy will positively affect user satisfaction with m-Health services.*

Effort expectancy is defined as “the degree of ease associated with the use of the system” [16] (p. 450). In this study, effort expectancy was conceptualized as the perceived ease of use of m-Health services. It has been found that security features increase customer trust in an online vendor and reinforce use intentions [29]. However, they can make it difficult for users to access the website and adversely affect user satisfaction. Consequently, users may experience greater satisfaction when they perceive m-Health applications to be easy to use during initial interactions. Accordingly, within the context of the ongoing COVID-19 pandemic in Taiwan (i.e., an emergency), we proposed the following hypothesis:

**Hypothesis** **2** **(H2).** *Effort expectancy will positively affect user satisfaction with m-Health services.*

Social influence has been defined as “the degree to which an individual perceives that important others believe he or she should use the new system” [16] (p. 451). Within the context of m-Health, social influence refers to the extent to which users are influenced by essential individuals (friends, family, colleagues, and supervisors) when they make decisions about using m-Health services. A study on mobile social applications found that social influence significantly affected user satisfaction and continuance intentions [26]. Similarly, Holden et al. [26] found that social influence (patients/families) directly influenced self-reported bar-coded medication administration satisfaction among nurses. It has been found that users are afforded several opportunities to recommend a particular service to others, but their recommendations depend on prior user satisfaction levels [30]. Thus, within the context of the ongoing COVID-19 pandemic in Taiwan (i.e., an emergency), the following hypothesis was proposed:

**Hypothesis** **3** **(H3).** *Social influence will positively affect user satisfaction with m-Health services.*

Facilitating conditions have been defined as “the degree to which an individual believes that an organization and technical infrastructure exist to support the use of the system” [16] (p. 453). Within the context of m-Health, facilitating conditions refer to user beliefs that technical support will be available to provide assistance and resolve technical issues to facilitate user adoption of m-Health applications. Festinger [31] found that, in some situations, facilitating conditions do not exert a substantial effect on satisfaction. As a result, users may negatively alter their behaviors. In contrast, Chan et al. [25] found that facilitating conditions significantly affect citizen satisfaction with e-government technology. Additionally, Hsiao and Tang [10] found that facilitating conditions constitute an important factor that directly influences m-Health technology adoption among older adults in Taiwan. Accordingly, within the context of the ongoing COVID-19 pandemic in Taiwan (i.e., an emergency), we proposed the following hypothesis:

**Hypothesis** **4** **(H4).** *Facilitating conditions will positively affect user satisfaction with m-Health services.*

Trust has been defined as the realization of expected but uncertain goals through reliance on an object’s characteristics, the occurrence of events, or the behaviors of individuals under conditions of risk [32]. Accordingly, Guo, Zhang, and Sun [33] found that private information has perceived safety and security increases user trust in m-Health services. Besides, Gefen, Benbasat, and Pavlou [34] found that trust is a crucial determinant of satisfaction among users toward technology. This finding indicates that user trust is influenced by experience. In their study, experienced users were more satisfied because they knew the ultimate outcomes of technology use.

In contrast, Pappas et al. [35] found that trust is an essential factor influencing user satisfaction among users with high and low levels of experience. Akter et al. [23] and Chiou [36] also found that perceived trust is an essential factor that positively influences user satisfaction and reinforces intentions to continue using m-Health services. Therefore, within the context of the ongoing COVID-19 pandemic in Taiwan (i.e., an emergency), we proposed the following hypothesis:

**Hypothesis** **5** **(H5).** *Trust will have a positive effect on user satisfaction with m-Health services.*

Satisfaction has been defined as the “feeling of pleasure or disappointment resulting from comparing a product’s perceived performance or outcome concerning his or her expectation” [37] (p. 153). Based on previous literature, Au, Ngai, and Cheng [38] found that satisfaction strongly influences user attitudes and intentions to use mBanking services. Sharma and Sharma [39] also found that satisfaction is a strong positive predictor of customer use intentions. In medical and m-Health services, Dagger et al. [40] and Säilä et al. [41] noted that patient satisfaction with hospitals is an essential factor that influences the effects of quality of service and service performance. Moreover, Barutçu et al. [42] found that m-Health user satisfaction has a positive effect on intentions to use m-Health services. Accordingly, within the context of the ongoing COVID-19 pandemic in Taiwan (i.e., an emergency), we proposed the following hypothesis:

**Hypothesis** **6** **(H6).** *User satisfaction will have a positive effect on m-Health emergency use intentions.*

Within the context of m-Health, use intentions refer to the extent to which individuals are positively disposed to use m-Health services [43]. Past studies have found that use intentions have a direct effect on actual usage behaviors and are an essential predictor of HIT adoption [9,16,22,44,45,46].

Emergency and routine use intentions are two different constructs that are equally important to research health services. According to Sundaram et al. [15], routine use intentions refer to integrating information technology into the daily routines of users, and are characterized by the attributes of repetitiveness, standardization, and regularization. In contrast, Liu et al. [13] noted that emergency use intentions are primarily specific to exigent circumstances (e.g., accidents). Further, in exigent situations, m-Health emergency use intentions are influenced by individuals’ unique healthcare needs. However, emergency use intentions have rarely been investigated [13]. Within the context of the ongoing COVID-19 pandemic, this study aimed to examine emergency use intentions (i.e., to use m-Health services) by focusing on individuals’ unique healthcare needs during medical emergencies. Therefore, we proposed the following hypothesis:

**Hypothesis** **7** **(H7).** *Emergency use intentions will have a positive effect on m-Health usage behaviors.*

Knowledge is related to “consumer expectations through ‘role understanding’, which is an antecedent to expectations and a mediator of the relationship between experience, familiarity, and expectations” [47] (p. 3). According to Rahman et al. [27], high levels of knowledge about m-Health services and greater access to mobile communication influence m-Health use intentions and usage behaviors.

However, few studies have examined the role of knowledge and mode of mobile phone use in healthcare in developing countries [27]. Moreover, the literature on the effect of m-Health knowledge on emergency use intentions is limited. We speculated that users who possess more technical knowledge would be more likely to report stronger m-Health use intentions. Thus, within the context of the ongoing COVID-19 pandemic in Taiwan (i.e., an emergency), the following hypothesis was proposed:

**Hypothesis** **8** **(H8).** *m-Health knowledge will have a positive effect on m-Health emergency use intentions.*

Age influences information technology adoption behaviors [16]. However, Chopra and Rajan [48] found that age does not significantly affect effort expectancy and use intentions. Venkatesh et al. [16] found that the effect of social influence on satisfaction increases with age. Chopra and Rajan [48] offered another unique perspective. Specifically, they observed that older adults tend to value their peers’, family members’, and friends’ opinions about the benefits of technology use. Besides, a past study found that older rather than younger salespersons were more likely to rely on training and support (facilitating conditions) to continue using technology [48]. However, the moderating role of age in m-Health adoption has rarely been investigated. Therefore, within the context of the COVID-19 pandemic in Taiwan (i.e., an emergency), the following hypotheses were proposed:

**Hypothesis** **9** **(H9).** *Age will moderate the relationships between (a) performance expectancy, (b) effort expectancy, (c) social influence, (d) facilitating conditions, and (e) trust and m-Health user satisfaction.*

Past studies have found that women and men hold different attitudes and behavioral intentions regarding decision-making situations related to the acceptance of new technology [16,49]. Gender has been found to play a significant role in technology use (e.g., m-Health services). Specifically, men report more positive perceptions of m-Health services than women [50]. In contrast, Yan [51] found that women are more likely to engage in health information-seeking behaviors than men. Accordingly, within the context of the ongoing COVID-19 pandemic in Taiwan (i.e., an emergency), we proposed the following hypotheses:

**Hypothesis** **10** **(H10).** *Gender will moderate the relationships between (a) performance expectancy, (b) effort expectancy, (c) social influence, (d) facilitating conditions, and (e) trust and m-Health user satisfaction.*

## 3. Materials and Methods

### 3.1. Survey Instrument

Quantitative data were collected using an online questionnaire. This questionnaire was originally developed in English, subsequently translated into Chinese, and then back-translated. A professional translator reviewed the questionnaire to ensure that the item contents were accurate translations. Additionally, the two English versions of the questionnaire were reviewed to ensure the consistency of survey items. A total of 34 questions were extracted from the literature and reworded (see Table 1). Items were rated on a 5-point Likert scale (1 = strongly disagree, 5 = strongly agree). Additionally, the questionnaire was evaluated by two experts from the healthcare industry; they assessed the ease of understanding, logical consistency, and sequence of items. They provided suggestions about the wording of several items, and the questionnaire contents were found to be clear and comprehensible. The online questionnaire was created using Google Forms, and the distribution was via the most used social media platforms in Taiwan (e.g., Line, Facebook, Instagram). The data were collected between 1–5 June 2020.

Before conducting the main study, the questionnaire was pilot tested on 30 respondents from Taiwan. The objective was to solicit additional feedback to improve item contents and the questionnaire as a whole. The authors made minor changes to some items and decided to provide a description of m-Health at the beginning of the questionnaire since the participants in the pilot test showed a lack of understanding of the term m-Health and the applications. These 30 pretest respondents were excluded from the main study in which tests of construct validity, scale reliability, and the proposed hypotheses were undertaken.

The questionnaire consisted of three sections (Appendix B). Before the participants could answer the questionnaire, they were provided with a description of m-Health services. The first section assessed prior experience and interaction with m-Health services. Next, the participants were divided into two groups based on their prior experience with m-Health services. The third section of the questionnaire assessed five demographic characteristics: gender, age, educational level, occupation, and monthly income. Three additional questions, which pertained to the COVID-19 quarantine parameters in Taiwan, were also included.

**Figure 1:**
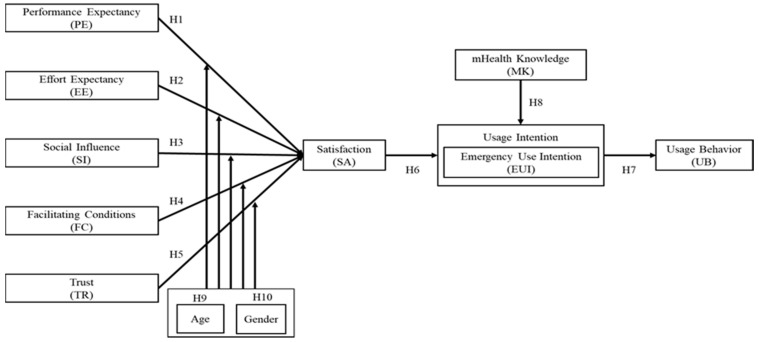
Research model.

This study aimed to extend the UTAUT model [16] by integrating three dimensions included in the UTAUT model: trust, satisfaction, and m-Health knowledge (Figure 1). These variables were assessed using the 34 items presented in Table 1.

### 3.2. Sampling Design and Data Collection

A total of 665 individuals participated in the study. Of these, 371 had used m-Health services during the past year. The data of only these individuals were analyzed. The response rate was 55.8 % (*N* = 371) following elimination of ineligible participants and those without prior experience with m-Health services. Participant demographic characteristics are presented in Table 2. Additionally, of these 371 respondents, 250 participants only used m-Health apps for COVID-19 mask, appointment and treatment-related matters, such as appointment services (*n* = 81), Mask map (*n* = 175), eMask (*n* = 151), NHI Express APP (*n* = 121) and PharmaCloud System (*n* = 17). In addition, 11 participants were in quarantine, 4 used m-Health services (e.g., Mask map, eMask, appointment services, NHI Express APP), 6 used face-to-face services, and 1 used emergency call to 119 (Table 2).

### 3.3. Data Analytic Procedure

Statistical Package for the Social Sciences (SPSS) version 25 was used to analyze the collected data. Additionally, AMOS version 20 was used to conduct structural equation modeling (SEM) and test the proposed research model (Figure 1). SEM was also used to test the moderating role of age and gender in performance expectancy, effort expectancy, social influence, facilitating conditions, trust, and satisfaction. To examine the latent variables’ validity and reliability, pooled confirmatory factor analysis (CFA) was conducted to estimate the measurement model [58]. Using AMOS, modification indices were generated. The final measurement model, which consisted of 28 items (original pool: 34 items), yielded acceptable model fit indices.

After pooled CFA was conducted, the structural model was tested across two stages. First, the effects of performance expectancy, effort expectancy, social influence, facilitating conditions, trust, and m-Health knowledge on usage behavior were examined. Next, we examined the moderating effects of age and gender by conducting a multi-group analysis. The sample (*N* = 371) was divided into three groups based on age (20–30 years, 31–50 years, and >51 years) and two groups based on gender (men and women) to independently examine the moderating effects of age and gender on the relationships, as mentioned earlier.

## 4. Results

**Figure A2:**
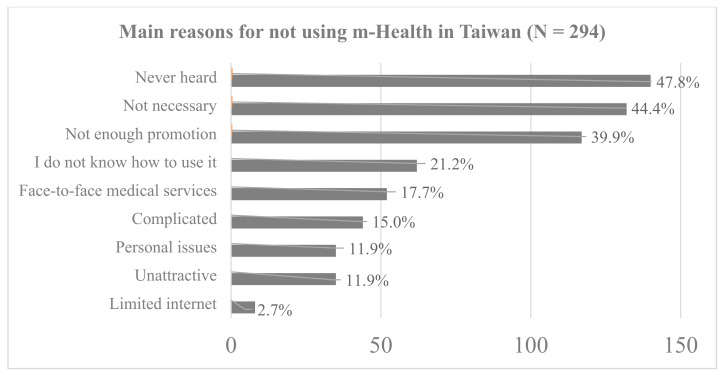
The reasons why people do not use m-Health in Taiwan.

To examine the ongoing pandemic’s impact, we identified the m-Health services that the participants most frequently used during the past three months (Appendix A). To gain a comprehensive understanding of these trends, we adopted the following premise: the effects of the COVID-19 pandemic are a principal contributor to m-Health use. Because the Taiwanese government controlled the sale and distribution of masks during the early stages of the pandemic, masks were sold at a fair price, and distribution was even [20]. Finally, this study explored why individuals fail to use m-Health services during the COVID-19 pandemic (Figure A2). Notably, some individuals in Taiwan are unfamiliar with the term “m-Health,” and individuals are mostly unfamiliar with m-Health services. However, compared to other countries, Taiwan has been vigilant and relatively stable in pandemic control. Indeed, no restrictions (e.g., lockdowns or quarantine regulations) were imposed. These factors appear to be one of the main reasons for the low demand for m-Health services during the COVID-19 pandemic.

### 4.1. Descriptive Statistics

We conducted pooled CFA to examine the multidimensionality, reliability, and validity of the proposed theoretical model. Based on the emergent factor loadings and squared multiple correlations, six items were deleted from the measurement model (social influence 3, facilitating conditions 1, trust 1, emergency use intentions 1, usage behavior 3 and m-Health knowledge 1). The loading of the six items that measured the latent variables was smaller than the recommended threshold of 0.6 [59]. The final pool of items (CFA indicators) and their factor loadings are presented in Table 3. The loadings of all the items that measured the latent variables were more significant than the recommended threshold of 0.6 [59]. Table 3 summarizes reliability (Cronbach’s α) and validity test results, including item loadings, construct reliability (CR) coefficients, and average variance extracted (AVE) values. Computing Cronbach’s alpha coefficients examined internal consistency. Cronbach’s alpha values >0.70 were considered to be acceptable [60]. Concerning reliability, all the measures’ CR values ranged from 0.71 to 0.93 and were more significant than the acceptable level of 0.60 [61]. The AVE values ranged from 0.46 to 0.81, but the value that emerged for facilitating conditions was lower than the recommended threshold of 0.5 [61]. According to Fornell and Larcker [61], the AVE value may be a more conservative estimate of a measurement model’s validity. Therefore, based on composite reliability alone, the researcher may conclude that “the convergent validity of the construct is adequate, even though more than 0.5 of the variance is due to error” (p. 46). In this study, the facilitating conditions factor’s CR value was more significant than the recommended threshold (0.5). Thus, the internal consistency based on items was considered to be acceptable. We inspected the item factor loadings to evaluate the convergent validity of the constructs. Each item loading was more significant than 0.6 and, therefore, considered to be adequately high [59]. The factor loadings of all the items ranged from 0.636 to 0.945 and exceeded the recommended level of 0.6 (see Table 3).

Researchers have recommended the following cutoff scores for model fit evaluations: χ^2^/df < 3 [62], AGFI > 0.8 [63], CFI > 0.9 [62], and RMSEA < 0.08 [64]. According to Doll, Xia, and Torkzadeh [65], GFI and AGFI values > 0.90 indicate good model fit, and values that fall between 0.80 and 0.89 indicate proper fit. The model fit indices yielded by the pooled CFA were used to evaluate construct validity.

Table 4 presents all the emergent model fit indices and the recommended thresholds. All the fit indices were within the recommended thresholds for good-fitting models. Thus, the results indicated that the model was a satisfactory fit for the collected data.

The values presented along the diagonal represent the square root of the AVE values of all constructs. These values were more generous than the correlation between the latent construct and any other construct. Table 5 presents the internal consistency coefficients, all of which were acceptable.

### 4.2. Structural Model and Hypotheses Testing

First, model fit indices were computed to evaluate the structural model. All the model fit indices met the recommended criteria (see Table 4). The analytic procedure included two steps. First, we examined the effects of performance expectancy, effort expectancy, social influence, facilitating conditions, and trust on usage behavior. Second, we investigated the moderating effect of age and gender. Additionally, we computed *p*-values to ascertain the significance of the results. Specifically, results with *p*-values < 0.05 were considered to be significant.

**Figure 2:**
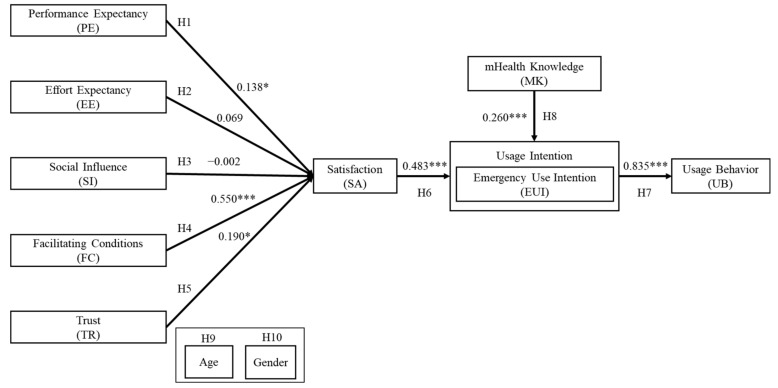
Results of the structural model. * *p* < 0.05, *** *p* < 0.001.

Figure 2 presents the results that emerged for the structural model. H1 and H4–H8 were supported, but H2 and H3 were not supported (Step 1). The hypothesis test results are shown in Table 6. The facilitating conditions factor was strongly associated with user satisfaction with m-Health services. Using bootstrapping, we found that user satisfaction partially mediated the effects of emergency use intentions on user behavior with m-Health services. Notably, Satisfaction and m-Health knowledge had a significant effect on emergency use intentions, which had a strong effect on user behavior.

### 4.3. Moderation Analysis

To explore the moderating role of age and gender (Step 2), the participants were divided into two groups. Table 7 presents the results of the moderation analysis. The results supported H9(a), H9(d), H9(e), H10(a), and H10(d); but not H9(b), H9(c), H10(b), H10(c), and H10(e).

The following variables’ effects were more substantial among 20–30-year-old participants: performance expectancy, facilitating conditions, and trust. Similarly, the effects of the following variables were stronger among women: performance expectancy and facilitating conditions.

## 5. Discussion

This study aimed to identify the key factors influencing user satisfaction, emergency use intentions, and usage behaviors concerning m-Health services in Taiwan. In this quantitative study, emergency use intentions significantly affected usage behaviors during the COVID-19 pandemic in Taiwan. Age and gender significantly moderated these effects. Of the 18 hypotheses that were proposed, 11 were supported by the results. The study findings are discussed in the following section.

This study indicates a lack of understanding of the term “m-Health and the applications,” despite the general use of mobile phone applications (such as medical appointments, mask distribution, etc.) for communicating health information. After the participants were provided with a description of m-Health services, they could understand the functionality and advantage that mobile health applications provide.

This study revealed that among the five predictors, performance expectancy, facilitating conditions, and trust had a significant effect on user satisfaction with m-Health services. Specifically, facilitating conditions had the most substantial effect on user satisfaction. It indicates that, among Taiwanese m-Health users, user satisfaction is most strongly influenced by the facilitating conditions created by m-Health service providers (e.g., service support, technical support). When users perceive m-Health service support to be satisfactory, they are likely to feel highly satisfied with m-Health services. Additionally, performance expectancy and trust emerged as important factors that influence m-Health user satisfaction. These results are consistent with past findings [11,23,24,25,28,31,36]. Because the Taiwanese government controlled the sale and distribution of masks during the early stages of the pandemic, masks were sold at a fair price, and distribution was even [20]. Therefore, in contrast to conventional mask distribution and location services, users perceived that mobile health services could provide more accurate service and new information on mask distribution services. Thus, we see that facilitating conditions and performance expectancy show a significant increase in users choosing m-Health services that better satisfy their needs.

Consequently, there was a significant positive correlation between trust and the behavior of patients in choosing services that better fulfill their satisfaction in the current study. Thus, optimal facilitating conditions, intense performance expectancy, and high levels of trust in m-Health service providers increase user satisfaction and reinforce emergency use intentions that benefit mobile health care technology and those applicable for COVID-19 disease.

In contrast, effort expectancy and social influence did not exert a significant effect. This finding indicates that effort expectancy and social influence are not significant determinants of user satisfaction among Taiwanese residents. In other words, the complexity or ease of use of m-Health services is not an essential, influential factor among Taiwanese m-Health users. These results contradict past findings [10,29]. Hsiao and Tang [10] found that facilitating conditions play an essential role in m-Health service acceptance among older adults in Taiwan. This may be the case because many older adults lack technical experience with new technology. Thus, the effect of effort expectancy may be stronger among older adults than among younger and middle-aged adults. However, the present sample consisted of individuals (*N* = 371) with prior experience with or knowledge about m-Health services.

Pappas et al. [35] found that the effect of effort expectancy on satisfaction was weaker among those with high levels of experience but more substantial among those with low experience levels. This discrepancy may be attributable to the socio-demographic characteristics of Taiwanese individuals. The social influence did not emerge as an essential factor that influences user satisfaction. The literature on this topic is ambivalent. Specifically, this observation is partially consistent with the findings of the studies conducted by Hsiao et al. [30] and Pappas et al. [35] but inconsistent with the findings of the studies conducted by Hsiao and Tang [10] and Shen and Chiou [29]. The findings are consistent with the results of studies that have examined the role of age. These studies have found that social influences play a more critical role in accepting new mobile technologies among young adults than among older adults [10,66]. However, in this study, social influence did not significantly affect, possibly because 70% of the participants were older than 30 years.

Satisfaction and m-Health knowledge had a positive effect on m-Health emergency use intentions. User satisfaction exerted the most potent effect, and the path coefficient was 0.483. This result is consistent with the studies conducted by [35,38]. The predictive relationship between satisfaction and m-Health use intentions, mainly emergency use intentions, has rarely been investigated in the past. In this study, m-Health knowledge had a weaker effect (0.260) on emergency use intentions than user satisfaction. Participants who possess m-Health knowledge may be more likely to use m-Health services in an emergency (Table 2). Our findings are consistent with the study results conducted by Rahman et al. [27] concerning the effect of knowledge on use intentions. However, few studies have examined the effect of knowledge on emergency use intentions. This study’s significant contribution pertains to the following observation: when individuals possess m-Health knowledge, they are willing to use it in emergencies because they recognize the benefits of m-Health services. In this study, satisfaction was strongly related to emergency use intentions. Thus, increasing user satisfaction by enhancing performance expectancies, facilitating conditions, and trust may be an effective means of reinforcing emergency use intentions. The government should increase public knowledge about m-Health services by conducting promotional campaigns and disseminating information. It will reinforce m-Health emergency use intentions among the general public in Taiwan.

In this study, m-Health usage behaviors were predicted by emergency use intentions. In other words, emergency use intentions had a significant effect (0.835) on user behavior. This result is consistent with past findings [13]. These results indicate that strong emergency use intentions motivate users to use m-Health services. Moreover, increasing user satisfaction and m-Health knowledge may be an effective means of promoting m-Health use in Taiwan.

Age and gender significantly moderated performance expectancy, facilitating conditions, and trust in user satisfaction with m-Health services. The moderating effect of age on the effects of all factors except performance expectancy, facilitating conditions, and trust was non-significant. Specifically, the effect of performance expectancy (0.268), facilitating conditions (0.523), and trust (0.236) on user satisfaction was stronger among 20–30-year-old participants than among the other age groups. This finding may be attributable to the fact that the younger participants grew up and received education in the digital age [16]. Younger individuals use mobile devices for a broader range of purposes (many of which simplify daily tasks and improve performance) than their older counterparts (e.g., to call or send a message). Thus, user perceptions may differ. In this study, the effects of performance expectancy and facilitating conditions on satisfaction were stronger among women than among men. However, there was no significant gender difference in the effect of effort expectancy, social influence, and trust on satisfaction. This indicates that gender differences in perception are not significant. Performance expectancy and facilitating conditions had a more substantial effect on satisfaction with m-Health services among women. Past studies have found that women and men hold different attitudes and engage in different behaviors when searching for health-related information. Thus, women may be more interested in acquiring health-related information than their male counterparts [51].

Additionally, according to a 2019 survey conducted by the NDC of Taiwan, rates of audiovisual learning, health, and other applications are higher among women than among men. The present findings indicate that performance expectancy and facilitating conditions positively affect user satisfaction among 20 to 30-year-old women in Taiwan. These observations are consistent with past research findings and survey results.

## 6. Implications

One of the challenges faced by m-Health managers pertains to how they can increase customer satisfaction to reinforce emergency use intentions. The present findings enhance our understanding of the successful implementation of m-Health services in Taiwan during the COVID-19 pandemic. Therefore, m-Health service developers in Taiwan should use the present findings to promote m-Health use among service users. As the proposed model suggests, user satisfaction increases when performance expectancy is high, facilitating conditions are optimal, and trust is strong. Therefore, m-Health service providers should offer services that fulfill user needs based on their psychological performance. However, more attention should be paid to facilitating conditions, which had the most potent effect on satisfaction with m-Health services. For example, exclusive emergency assistance functions should be provided to quarantined individuals during the pandemic. In this manner, the Taiwanese government can disseminate information about m-Health services among citizens to increase m-Health usage behaviors. Mobile health is helping to solve important challenges in the healthcare system, including the lack of efficiency and the unnecessary healthcare waste. This m-Health solutions helps policymakers and health providers contain the spread of epidemics through m-Health services. Thus, is critical to understand the adoption of m-Health services in the context of the COVID-19 pandemic.

Moreover, user satisfaction exerted the strongest effect. Therefore, m-Health providers need to offer satisfactory services to reinforce emergency use intentions. Furthermore, increasing m-Health knowledge is a vital user retention strategy. Thus, to reduce the spread of diseases and reduce person-to-person contact, the government should educate individuals about using m-Health services through television advertisements and encourage them to use these services. Besides, m-Health service providers should pay attention to their service users’ use intentions and promote usage behaviors. In particular, they should reinforce emergency use intentions by providing timely and useful functionalities and creating a more user-friendly interface.

The effect of performance expectancy and facilitating conditions was more substantial among young women, and the effect of trust on user satisfaction was more substantial among young adults. The number of m-Health service users in Taiwan increased by more than 3 million during the COVID-19 pandemic. Compared to the previous year, this translates to an increase of 66% within only a few months [67]. Therefore, m-Health service providers should offer different product features and services based on user age and gender to enhance user satisfaction and provide high-quality services to different users. Moreover, the government should implement different supportive measures for men and those older than 30 years to enhance their satisfaction with m-Health services.

## 7. Conclusions

This study aimed to extend the UTAUT model by focusing on adopting m-Health services during the COVID-19 pandemic in Taiwan (an emergency). This research is one of the first investigations to have examined the adoption of m-Health services during the COVID-19 pandemic. m-Health was used to analyze mask distribution, medical appointments, and mobile health consultation of patients on quarantine during the COVID-19 pandemic in Taiwan. This study makes significant theoretical contributions to the literature on m-Health adoption during the COVID-19 pandemic in Taiwan. This study identified the factors (i.e., performance expectancy, facilitating conditions, and trust) that have positive effects on user satisfaction with m-Health services, which influenced the adoption of m-Health services during the COVID-19 pandemic. User satisfaction, emergency use intentions, and m-Health knowledge had positive effects on m-Health usage behaviors. These findings offer useful insights that can be used to increase user satisfaction and m-Health service use in Taiwan. Thus, this study contributes to the literature on m-Health adoption. Notably, the findings underscore the key variables that influence satisfaction, emergency use intentions, and m-Health usage behaviors during the COVID-19 pandemic. In this study, user satisfaction and m-Health knowledge had strong effects on emergency use intentions. Besides, we also explored user perceptions of m-Health emergency use intentions in Taiwan. It is an area that has rarely been explored. Therefore, our findings enhance our understanding of HIT applications, which are undergoing rapid development.

The most apparent managerial implication of the present findings is that optimal facilitating conditions will significantly increase user satisfaction among Taiwanese m-Health users. Concerning the moderating role of age and gender, we found that satisfaction was more strongly influenced by higher levels of performance expectancy, facilitating conditions, and trust among young women than among their male counterparts. Thus, enhancing performance expectancy, optimizing facilitating conditions, and increasing user trust will promote m-Health use among young women. Moreover, we believe that no past study has explored the use of m-Health services during the COVID-19 pandemic in Taiwan.

### Limitations and Recommendations

This study has some limitations, which should be acknowledged. In this quantitative research study, which was conducted during a public health emergency (i.e., the COVID-19 pandemic), m-Health emergency use intentions and usage behaviors were examined by conducting comprehensive analyses and using a representative sample. However, m-Health use is a complex process that is influenced by several factors. Therefore, in-depth investigations into m-Health use should be undertaken by conducting qualitative interview studies to understand the most critical issues faced by m-Health service users. Second, this study adopted a cross-sectional survey design. Thus, experience, emergency use intentions, and usage behaviors were measured only at a single time point (i.e., during the COVID-19 pandemic). Thus, contrary to the most primitive UTAUT study, this study uses a longitudinal survey and lacks a comparison of the time before and after the use intentions of m-Health services in Taiwan.

To extend the present findings, future studies should identify additional influential factors. Notably, the moderating effect of m-Health knowledge should be examined. This study was conducted among users in Taiwan. Hence, the results may be attributable to cultural factors. Therefore, future studies should recruit samples from both Eastern and Western countries. In this study, data were collected using an online survey questionnaire. Therefore, only users with internet access could participate. In future studies, pen-and-paper questionnaires should also be made available to enhance the survey results’ validity.

## Figures and Tables

**Table 1:** Constructs and measurement items.

Constructs	Items	Description	Item Source
Performance Expectancy	PE1	I find m-Health useful in my life during the COVID-19 pandemic.	[16,22].
	PE2	Using m-Health increases my chances of meeting my needs during the COVID-19 pandemic.	
	PE3	Using m-Health helps me in manage my daily healthcare during the COVID-19 pandemic.	
	PE4	Using m-Health service increases my capability to manage my health during the COVID-19 pandemic.	
Effort Expectancy	EE1	Learning how to use m-Health is easy for me.	[16,22].
	EE2	My interaction with m-Health is clear and understandable.	
	EE3	I find m-Health easy to use.	
Social Influence	SI1	People who are important to me think that I should use m-Health services during the COVID-19 pandemic.	[16,22].
	SI2	People who influence my behavior think that I should use m-Health during the COVID-19 pandemic.	
	SI3	People in my social groups who use m-Health service are seen as more prestigious than those who do not.	
Facilitating Conditions	FC1	I have the resources necessary to use m-Health services.	[16,22].
	FC2	I have the knowledge necessary to use m-Health services.	
	FC3	I can get help from others when I have difficulties using m-Health services.	
	FC4	m-Health instructions are useful to me when I use m-Health services.	
Trust	TR1	I trust my m-Health applications during the COVID-19 pandemic.	[52,53,54].
	TR2	I find m-Health reliable in conducting health services during the COVID-19 pandemic.	
	TR3	I feel that m-Health is safe for receiving reliable medical information during the COVID-19 pandemic.	
	TR4	I trust m-Health’s commitment to satisfy my medical information needs during the COVID-19 pandemic.	
Satisfaction	SA1	I am satisfied with m-Health efficiency.	[55,56].
	SA2	I am satisfied with m-Health service quality.	
	SA3	I am satisfied with the presentation of the m-Health service’s user interface.	
	SA4	I am satisfied with my overall experience using m-Health.	
Emergency use intention	EUI1	I use m-Health services when I am in urgent need of medical care during the COVID-19 pandemic.	[13,16].
	EUI2	I will consider using m-Health services if I have urgent medical requirements during the COVID-19 pandemic.	
	EUI3	m-Health is the first choice if I need urgent medical health services during the COVID-19 pandemic.	
	EUI4	I will continue to use m-Health services if I need urgent medical care in future.	
Usage Behavior	UB1	m-Health service is a pleasant experience.	[16,22].
	UB2	I really want to use m-Health services to keep my healthy during the COVID-19 pandemic.	
	UB3	I spend a lot of time using m-Health services during the COVID-19 pandemic.	
	UB4	I use m-Health services on regular basis during the COVID-19 pandemic.	
m-Health Knowledge	MK1	I have already used or practiced m-Health Apps to make myself familiar with the functionality.	[57].
	MK2	I have already made myself familiar with different versions of m-Health.	
	MK3	I know the types of m-Health Apps that are commonly used.	
	MK4	I can skillfully operate commonly used m-Health Apps.	

**Table 2:** Sample characteristics.

Demographic Characteristics	Have used m-Health (*N* = 371) 55.8%		Have not used m-Health (*N* = 294) 44.2%	
	Frequency	%	Frequency	%
**Gender**				
Male	173	46.6	144	49.0
Female	198	53.4	150	51.0
**Age**				
20–30 years	111	29.9	111	37.8
31–50 years	175	47.2	101	34.4
More than 51 years	85	22.9	82	27.9
**Education**				
Below senior high school	40	10.8	55	18.7
University	206	55.5	175	59.5
Above of Master	125	33.7	64	21.8
**Profession**				
Employee	295	79.5	203	69.1
Student	55	14.8	60	20.4
Home keeper	21	5.7	31	10.5
**About COVID-19**				
**(a) Home quarantine**				
Yes	11	3.0	6	2.0
No	360	97.0	288	98.0
**(b) Quarantine place**	(*N* = 11)		(*N* = 6)	
Home	9		5	
Epidemic Prevention Hotel	2		1	
**(c) Priority medical service**	(*N* = 11)		(*N* = 6)	
m-Health services or Apps	6		1	
Face-to-face service	4		3	
Emergency call 119	1		2	
**(d) Use of m-Health APP related to COVID-19 in the past three moths**	(*N* = 665)		(*N* = 665)	
Yes	371	55.8		
No			294	44.2%

**Table 3:** Standard item loadings, Cronbach’s α, Composite Reliability, and Average Variance Extracted.

Factors	Items	Mean	Factor Loadings	CR	AVE	Cronbach’s α
Performance Expectancy	PE1	4.13	0.689	0.881	0.651	0.877
	PE2	4.08	0.844			
	PE3	4.04	0.846			
	PE4	3.98	0.837			
Effort Expectancy	EE1	4.02	0.823	0.925	0.806	0.924
	EE2	3.92	0.945			
	EE3	3.95	0.920			
Social Influence	SI1	3.65	0.885	0.860	0.754	0.859
	SI2	3.47	0.851			
Facilitating Conditions	FC2	3.94	0.731	0.714	0.455	0.715
	FC3	3.92	0.652			
	FC4	3.91	0.636			
	TR2	3.91	0.813			
Trust	TR3	3.97	0.845	0.870	0.690	0.869
	TR4	3.95	0.833			
Satisfaction	SA1	3.90	0.867	0.919	0.740	0.915
	SA2	3.91	0.901			
	SA3	3.73	0.785			
	SA4	3.91	0.883			
Emergency use intention	EUI2	3.77	0.819	0.906	0.763	0.904
	EUI3	3.56	0.893			
	EUI4	3.72	0.906			
Usage Behavior	UB1	3.89	0.849	0.839	0.635	0.834
	UB2	3.78	0.779			
	UB4	3.74	0.759			
m-Health Knowledge	MK2	3.32	0.794	0.887	0.725	0.886
	MK3	3.56	0.880			
	MK4	3.54	0.877			
Total						0.865

**Table 4:** Model fit indices and target values in CFA and SEM.

Quality-of-Fit Measure	Recommended Value	Measurement Model (CFA)	Structural Model (SEM)
χ^2^		583.328	884.488
d.f		314	327
χ^2/d.f^	<3.00	1.858	2.705
*p*-Value		0.000	0.000
GFI	>0.80	0.901	0.858
AGFI	>0.80	0.872	0.824
CFI	>0.90	0.966	0.930
RMSEA	<0.07	0.048	0.068

**Table 5:** Descriptive statistics and correlation matrix.

	CR	AVE	PE	EE	SI	FC	TR	SA	EU	UB	MK
PE	0.881	0.650	0.806								
EE	0.925	0.806	0.507 **	0.897							
SI	0.860	0.754	0.461 **	0.412 **	0.868						
FC	0.714	0.455	0.652 **	0.580 **	0.457 **	0.674					
TR	0.877	0.642	0.652 **	0.580 **	0.457 **	0.635 **	0.801				
SA	0.919	0.740	0.615 **	0.657 **	0.471 **	0.640 **	0.688 **	0.860			
EU	0.906	0.764	0.528 **	0.414 **	0.481 **	0.469 **	0.599 **	0.547 **	0.874		
UB	0.839	0.635	0.598 **	0.536 **	0.551 **	0.587 **	0.660 **	0.731 **	0.701 **	0.797	
MK	0.887	0.725	0.447 **	0.564 **	0.467 **	0.582 **	0.525 **	0.655**	0.498 **	0.672 **	0.851

Note. *N* = 371; diagonal elements are the square root of AVE; ** *p* < 0.01.

**Table 6:** Hypothesis testing.

Hypothesis	Path	Standardized Path Coefficient	*t*-Statistics	Test Result
H1	PE→SA	0.138 *	2.076	Supported
H2	EE→SA	0.069	0.882	Not Supported
H3	SI→SA	−0.002	−0.038	Not Supported
H4	FC→SA	0.550 ***	3.939	Supported
H5	TR→SA	0.190 *	2.004	Supported
H6	SA→EUI	0.483 ***	7.350	Supported
H7	EUI→UB	0.835 ***	14.030	Supported
H8	MK→EUI	0.260 ***	4.038	Supported

* *p* < 0.05, *** *p* < 0.001.

**Table 7:** Moderating effect of age and gender.

Hypothesized Paths	(H9) Age			(H10) Gender			
	20–30 Years Old	31–50 Years Old	More Than 51	Support	Male	Female	Support
	(*N* = 111)	(*N* = 175)	(*N* = 85)		(*N* = 173)	(*N* = 198)	
(a) PE→SA	0.268 *	0.219 *	0.174 *	Partial	0.146 *	0.196 *	Partial
(b) EE→SA	0.042	0.035	0.037	Not	0.063	0.072	Not
(c) SI→SA	−0.010	−0.009	−0.009	Not	0.051	0.056	Not
(d) FC→SA	0.523 *	0.515 *	0.429 *	Partial	0.595 *	0.638 *	Partial
(e) TR→SA	0.236 *	0.204*	0.182*	Partial	0.109	0.110	Not

* *p* < 0.05.

**Figure A1:**
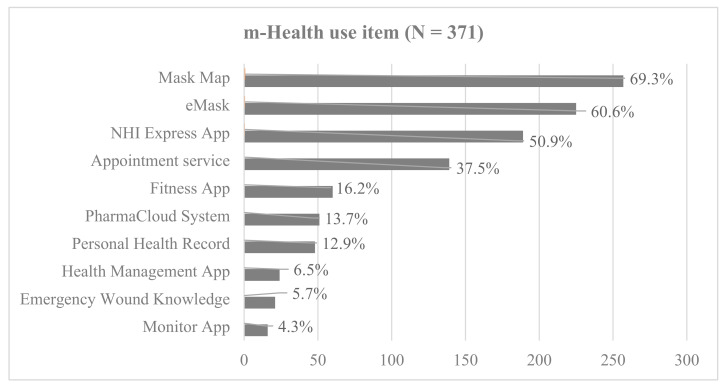
m-Health services used in Taiwan.

## Data Availability

Data sharing not applicable.

## Appendix B

Questionnaire

Mobile health (m-Health) definition: “m-Health” is defined as “medical and public health practice supported by mobile devices, such as mobile phones, patient monitoring devices, personal digital assistants (PDAs), and other wireless devices” (for example information about covid-19 as well as other services related to the health care system) (World Health Organization (WHO)).

m-Health application for example:

1. Appointment Service
2. Mask Map
3. eMask
4. NHI Express APP
5. PharmaCloud System
6. Fitness App
7. Health Management App
8. Monitor App
9. Personal Health Record
10. Emergency Wound Knowledge

Part one

Experience	
1. Have you heard of m-Health related information before answering the questionnaire?	Yes/No
2. After hearing the m-Health introduction above, do you clearly understand the content of m-Health?	Yes/No
3. In the past three months, have you checked for information about” COVID-19” using any App?	Yes/No
4. In the past three months, have you used m-Health services or any health App?	Yes/No
4-1 Which option is the reason why you have not used an "m-Health Mobile Medical" service or application? (more than one option) □Limited internet service on my phone □There is not necessary □Never heard about it □I do not know how to use it □Not enough promotion □It is too complicated □I prefer face-to-face medical services □Unattractive □Personal issues □Other reason	
5. In the past three months, which of the following m-Health services or Apps have you used? (more than one option) □Appointment service □Mask Map □eMask □NHI Express App □PharmaCloud System □Fitness App □Health Management App □Monitor App □Personal Health Record □Emergency Wound Knowledge □Other	
6. In the past three months, how often have you used any m-Health services or Apps? □ Daily □ Once a week □ Every two weeks □ Once a month	

Part two (Likert 5-point)

Performance Expectancy
1. I find m-Health useful in my life during this pandemic COVID-19 situation. 2. Using m-Health increases my chances of meeting my needs during this pandemic COVID-19 situation. 3. Using m-Health helps me in manage my daily healthcare during this pandemic COVID-19 situation. 4. Using m-Health service increases my capability to manage my health during this pandemic COVID-19 situation.
Effort expectancy
1. Learning how to use m-Health is easy for me. 2. My interaction with m-Health is clear and understandable. 3. I find m-Health easy to use.
Social influence
1. People who are important to me think that I should use m-Health services during this pandemicCOVID-19 situation. 2. People who influence my behavior think that I should use m-Health during this pandemic COVID-19 situation. 3. People in my social groups who use m-Health service are seen as more prestigious than those who do not.
Facilitating conditions
1. I have the resources necessary to use m-Health services. 2. I have the knowledge necessary to use m-Health. 3. I can get help from others when I have difficulties using m-Health services. 4. m-Health instructions are useful to me when I use m-Health services.
Trust
1. I trust my m-Health applications during this pandemic COVID-19 situation. (e.g., usage of anonymous personal data and information). 2. I find m-Health reliable in conducting health services during this pandemic COVID-19 situation. (e.g., appointment and consult). 3. I feel that m-Health is safe for receiving reliable medical information during this pandemic COVID-19 situation. 4. I trust m-Health’s commitment to satisfy my medical information needs during this pandemic COVID-19 situation.
Satisfaction
1. I am satisfied with m-Health’s efficiency. 2. I am satisfied with m-Health’s service quality. 3. I am satisfied with the presentation of the m-Health service’s user interface. 4. I am satisfied with my overall experience using m-Health.
Emergency use intention
1. I use m-Health services when I am in urgent need of medical care during this pandemic COVID-19 situation. 2. I will consider using m-Health services if I have urgent medical requirements during this pandemic Covid-19 situation. 3. “m-Health” is the first choice if I need urgent medical health services during this pandemic Covid-19 situation. 4. I will continue to use m-Health services if I need urgent medical care in future.
Usage behavior
1. m-Health service is a pleasant experience. 2. I really want to use m-Health services to keep my healthy during this pandemic COVID-19 situation. 3. I spend a lot of time using m-Health services during this pandemic COVID-19 situation. 4. I use m-Health services on regular basis during this pandemic COVID-19 situation.
m-Health Knowledge
1. I have already used or practiced m-Health App’s to make myself familiar with the functionality. 2. I have already made myself familiar with different versions of m-Health. 3. I know the types of m-Health Apps that are commonly used. 4. I can skillfully operate commonly used m-Health Apps.

Part three

Demographics
1. Gender: 口male 口 female
2. Age: 口20(include)-30 口31~40 口41~50 口51~65口more than 65
4. Education: 口Below junior high school(include) 口Senior high school (include vocational) 口University 口Above of Master
5. Income: 口NT$10,000 or less 口NT$10,001~30,000 口NT$30,001~50,000口NT$50,001~70,000 口NT$70,001 or more
6. In the past, have you been asked to complete home isolation or quarantine? 口yes 口no
7. Where have you been quarantined in the past? 口Home口Epidemic Prevention Hotel
8. During quarantine, which medical service did you prioritize for any medical requirements? 口m-Health services or apps 口Face to face service 口Emergency call 11
